# A single-center investigation on serotypes, drug resistance and clinical significance of GBS isolates from pregnant and non-pregnant adults in Baoji, China

**DOI:** 10.3389/fcimb.2025.1556603

**Published:** 2025-07-01

**Authors:** Siyu Chen, Haiying Li, Mengyang Guo, Hua Wang, Wei Gao, Yating Cui, Yani Zhang, Lin Yuan, Wei Shi, Kaihu Yao

**Affiliations:** ^1^ Ministry of Education Key Laboratory of Major Diseases in Children, Beijing Key Laboratory of Pediatric Respiratory Infection Diseases, Beijing Pediatric Research Institute, Beijing Children’s Hospital, Capital Medical University, National Center for Children’s Health, Beijing, China; ^2^ Department of Laboratory Medicine, Baoji People’s Hospital, Baoji, Shannxi, China

**Keywords:** group B streptococcus, adults, infection, serotype, antimicrobial resistance

## Abstract

**Objectives:**

To assess the epidemiology, serotype distribution, and antimicrobial resistance of GBS among both pregnant and non-pregnant adults in Baoji, China, addressing an existing gap in current research.

**Methods:**

Based on the GBS strains identified in the clinical laboratory from 2016 to 2024, information on age, gender, specimen type, and diagnosis was collected for the corresponding adult cases, including both colonization and infection cases. GBS was identified using three methods: mass spectrometry, CAMP test and latex agglutination kit for streptococcal serotyping. Serotypes were determined by latex agglutination, and antimicrobial susceptibility was tested against 20 antimicrobials using an automated drug susceptibility system.

**Results:**

A total of 200 GBS strains were collected, in which 107 were from pregnant women, and 93 from non-pregnant adults including 34 males. Clinical pathogenic isolates were defined for 86 cases, in which the urinary tract infections were predominant (61.6%), and invasive infections were confirmed for 16 cases (18.6%). A total of 5 serotypes were identified in the present 200 strains, including serotype Ib (34.5%), V (26.0%), III (21.5%), Ia (2.0%) and VIII (1.0%). In addition, 30 strains (15.0%) were non-typeable (NT). The coverage rate of the hexavalent vaccine currently in development is 84.5%. Significant differences are observed in the proportions of serotypes Ib, III, and/or V across various age groups, pregnancy statuses, and between colonized and infectious strains. Notably, the proportion of serotype V varies markedly, with 32.3% in the 18–39 age group versus 18.9% in the 40–64 age group and 10% in the >64 age group, 36.4% in pregnant women compared to 14.0% in non-pregnant women, and 34.2% in colonized strains as opposed to 15.1% in infectious ones. All 200 GBS strains were sensitive to penicillin, and the resistance rates to erythromycin, azithromycin, levofloxacin, clindamycin, tetracycline, and chloramphenicol were 93.5%, 93.5%, 70.5%, 70.0%, 53.5%, and 13.0%, respectively.

**Conclusion:**

The present findings suggest that GBS may potentially infect and colonize adults, regardless of gender or age, in Baoji, China. Serotypes Ib, III, and V are common serotypes, but their frequency is related to the host’s age group, pregnancy status, and clinical relevance. GBS isolates are still generally susceptible to penicillin.

## 1 Introduction

Group B streptococcus (GBS), also known as *Streptococcus agalactiae*, is the main causative agent of neonatal sepsis and meningitis. Neonatal GBS infections can be categorized as early onset disease (EOD) and late onset disease (LOD), defined as infections occurring between 0–6 day and 7 day-3 months postpartum, respectively (Chinese Society of Perinatal Medicine, Obstetrics Group of Chinese Society of Obstetrics and Gynecology, 2021). It is commonly believed that neonates are infected with GBS through vertical transmission from mother to child. Developed countries such as the United States and the United Kingdom have recommended the use of intrapartum antibiotic prophylaxis (IAP) to reduce the burden of GBS disease in neonates. However, the implementation of IAP is effective in reducing GBS-EOD only without impact on GBS-LOD (Russell et al., 2017). This may be related to the fact that the source of bacterial infection in LOD is not limited to mothers merely (Jauneikaite et al., 2018; Freudenhammer et al., 2021; Miselli et al., 2022). Numerous epidemiological studies have focused on the colonization and infection of GBS in perinatal populations, including pregnant women and neonates. In contrast, investigations of GBS infections in non-pregnant adults are relatively scarce, suggesting that such infections may be less common in this population. In China, GBS infection in non-pregnant adults has rarely been studied. However, it has long been shown that GBS is associated with invasive infections in adults, especially in groups > 65 years of age and those with underlying diseases (Edwards and Baker, 2005). The incidence of invasive GBS in the non-pregnant U.S. population is on a steady rise (Francois Watkins et al., 2019). Martins et al. indicated that the rate of GBS colonization in non-pregnant adults increased with age (18-29-year-olds and 30-44-year-olds: ~25%; ≥60 year old group: >42%), suggesting that the prevalence of GBS infection may also increase with age (Martins et al., 2022). To uncover the facts of GBS infection in Chinese adults, all isolates of GBS from adults (including pregnant and non-pregnant women) were cultured and identified. The specimen type, corresponding case demographic information and diagnosis were reviewed in medical records. The phenotypic characteristics of these isolates, including serotype and drug sensitivity, would be analyzed and discussed in this study, addressing a gap in current research.

## 2 Materials and methods

### 2.1 GBS isolates and identification

**Figure 1:**
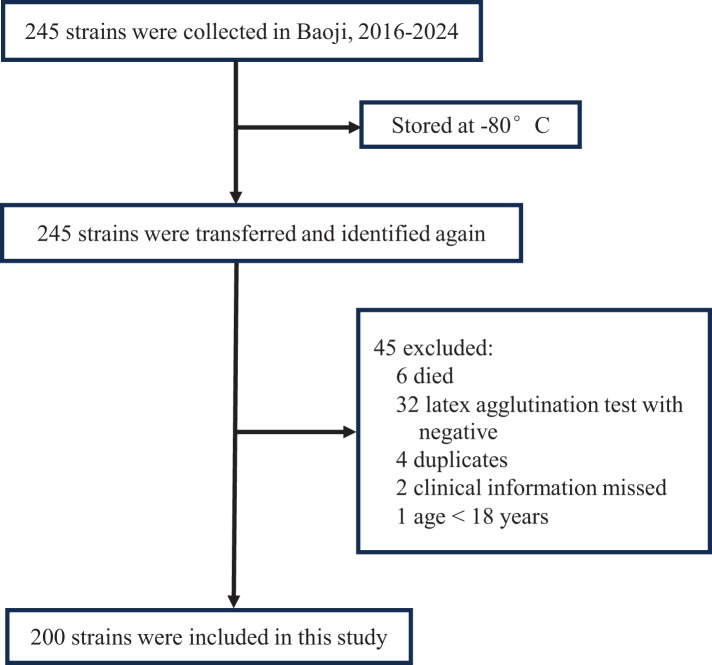
The collection ways of the GBS strains included in the present study.

Between 2016 and 2024, clinical isolates identified as GBS using the VITEK mass spectrometer (BIOMERIEUX, France) were stored at ultra-low temperatures (−80°C) at the clinical laboratory of Baoji People’s Hospital. For this study, the stored strains were revived and cultured in Tryptic Soy Agar (TSA) supplemented with 5% sheep blood. The latex agglutination streptococcal grouping kit (Streptococcal grouping kit, OXOID, UK) and the Christie-Atkins-Munch-Petersen (CAMP) test were employed to confirm GBS identification. Strains that tested positive by both methods were included in the study (**Figure 1**). In parallel, clinical data were retrospectively collected for the corresponding patients, including gender, age, sample source, and clinical diagnosis. After excluding duplicate samples, all samples from individuals aged over 18 were included in the analysis.

### 2.2 Clinical information

We browsed the GBS-related literature and clinical diagnostic guidelines to identify the types of infections known to be caused by GBS as well as the criteria that define invasive GBS disease (Le Doare and Heath, 2013; Francois Watkins et al., 2019; Manuel et al., 2024). At the same time, we also reviewed patients’ clinical diagnostic records. Clinical pathogenic isolates were identified when GBS strains matched predefined infection criteria from established clinical guidelines. The criteria for defining invasive GBS disease include the isolation of GBS from normally sterile sites, such as blood, abdominal fluid, pleural fluid, joint fluid, bronchoalveolar lavage, drainage fluid, cerebrospinal fluid, amniotic fluid, peritoneal fluid, and the placenta. Carriage isolates were defined as strains from non-sterile anatomical sites, absence of guideline-supported infection criteria, no clinical manifestations of active disease.

### 2.3 GBS serotyping

The rapid latex agglutination test was used to identify the serotypes of GBS isolates, covering ten serotypes, including Ia, Ib, and II-IX. The Latex Agglutination Assay Kit was purchased from Statens Serum Institute (SSI), Denmark. Strains that did not agglutinate with any of the ten antisera were classified as non-typable (NT). Specific procedures and result interpretations were conducted according to the kit’s instruction manual.

### 2.4 Antimicrobial drug susceptibility analysis

Using Sensititre™ ARIS™ 2X fully automated drug sensitivity assay (Thermo Fisher Scientific, USA) and Thermo Fisher Streptococcus drug sensitivity plate (B4073A), 20 antimicrobial drugs were tested: vancomycin (0.5–4 mg/L), clindamycin (0.12–1 mg/L), linezolid (0.25–4 mg/L), ertapenem (0.5–4 mg/L), tetracycline (1–8 mg/L), cotrimoxazole (0.5/9.5-4/76 mg/L), cefepime (0.5–8 mg/L), cefuroxime sodium (0.5–4 mg/L), cefotaxime (0.12–4 mg/L), ceftriaxone (0.12–2 mg/L), levofloxacin (0.5–4 mg/L), tigecycline (0.015-0.12 mg/L), chloramphenicol (1–32 mg/L), erythromycin (0.25–2 mg/L), meropenem (0.25–2 mg/L), moxifloxacin (1–8 mg/L), daptomycin (0.06–2 mg/L), azithromycin (0.25–2 mg/L), amoxicillin/clavulanic acid (2/1-16/8 mg/L), penicillin (0.03–4 mg/L). Streptococcus pneumoniae ATCC49619 was the quality control strain.

Outcome determination was based on the 2024 American Clinical and Laboratory Standards Institute (CLSI) standards. In case of penicillin non-susceptible strains, we will use the E-test for re-verification. The E-test strips were purchased from BIOMERIEUX, France.

### 2.5 Vaccine coverage rate

The Pfizer GBS6 vaccine currently in Phase 2 clinical trials (NCT03765073) targets six major GBS capsular polysaccharide serotypes (Ia, Ib, II, III, IV, and V), which account for 98% of GBS disease worldwide (Madhi et al, 2023). We analyzed the distribution of the six serotypes (Ia, Ib, II, III, IV, and V) in our study population and estimated the vaccine coverage based on this distribution: Vaccine coverage= (Serotype prevalence in this study/Serotypes included in the vaccine) × 100%.

### 2.6 Statistical analysis

Count data were presented as the number of cases or strains and their corresponding rates. Comparisons between groups were statistically analyzed using the χ2 test or Fisher’s exact test, as appropriate. The Cochran-Armitage trend test was employed for trend analysis. A p-value of less than 0.05 was considered statistically significant.

## 3 Results

### 3.1 Demographic and clinical characteristics

During the study period, a total of 245 GBS strains were stored in the local clinical laboratory. After undergoing re-culture and identification, a total of 200 GBS strains were ultimately included in this study.

The median age of the corresponding 200 patients was 33 years (IQR: 29-57). There were 107 (53.5%) pregnant women and 93 (46.5%) non-pregnant adults including 34 males. The 200 strains were isolated respectively from urine (n=73), vaginal secretions (n=100), localized lesion secretions (n=10), amniotic fluid (n=7), blood (n=6), fetal membranes swabs (n=3), and breast milk (n=1). According to the present classification criteria, 86 strains were clinical pathogenic isolates and the other 114 ones were clonal or carriage isolates. The major relative clinical diagnosis of the 86 patients included urinary tract infection (n=53), vaginitis (n=9), skin and soft tissue infection (n=7), bacteremia (n=6), chorioamnionitis (n=10), mastitis (n=1). Among the current carrier isolates (n = 114), 79.8% (n = 91) were obtained from vaginal swabs of pregnant women during routine prenatal screening, whose median age was 29 years (IQR: 25-32). The remaining 23 isolates were recovered from urine samples, comprising 10 from males and 13 from non-pregnant females, whose median age was 61 years (IQR: 29-71). GBS colonizes the reproductive tract predominantly in women of childbearing age, while GBS infections are more prevalent in the middle-aged and older age groups, and infections are predominantly urinary tract infections. (**Table 1**). A total of 16 strains were identified as invasive GBS infection during the 9-year study period, which included 10 chorioamnionitis cases and 6 bacteremia or septicemia diseases. The median age of these 16 patients was 33.5 years (IQR: 29.25-60). The six patients with bacteremia aged from 53 to 85, four of whom were male. In the cohort of 10 patients with chorioamnionitis (median age 30 years; IQR: 29-32), specimens were isolated from amniotic fluid in seven cases and from fetal membranes in three cases, all collected during caesarean section.

**Table 1:** Demographic characteristics, specimen sources, and clinical characteristics of the present 200 GBS isolates, 2016–2024 [n (%)].

Characteristic	Total (n=200)	Clinical pathogenic isolates (n=86)	Carriage isolates (n=114)	P values
Sex				<0.001
Male	34 (14.0)	24 (27.9)	10 (8.8)	
Female	166 (83.0)	62 (72.1)	104 (91.2)	
Age (years)				<0.001
18-39	127 (63.5)	32 (37.2)	95 (83.3)	
40-64	43 (21.5)	33 (38.4)	10 (8.8)	
>64	30 (15.0)	21 (24.4)	9 (7.9)	
Pregnancy^a^	166 (83.0)	62 (72.1)	104 (91.2)	<0.001
Pregnant	107 (53.5)	16 (18.6)	91 (79.8)	
Non-pregnant	59 (29.5)	46 (53.5)	13 (11.4)	
Isolation sites				<0.001
Urine	73 (36.5)	50 (58.1)	23 (20.0)	
Vagina	100 (50.0)	9 (10.5)	91 (79.8)	
Local lesion	10 (5.0)	10 (11.6)	0 (0.0)	
Blood	6 (3.0)	6 (7.0)	0 (0.0)	
Amnion cavity^b^	10 (5.0)	10 (11.6)	0 (0.0)	
Milk	1 (0.5)	1 (1.2)	0 (0.0)	
Clinical manifestations				
UTI^c^		53 (61.6)		
SSTI^d^		7 (8.1)		
Vaginitis		9 (10.5)		
Invasive infection		16 (18.6)		
Mastitis		1 (1.2)		
Total	200 (100.0)	86 (43.0)	114 (57.0)	

Pregnancy^a^: only females are included; Amnion cavity^b^: 7 amniotic fluid, 3 fetal membranes swabs, all were isolated during caesarean section; UTI^c^: urinary tract infection; SSTI^d^: Skin and soft tissue infection.

### 3.2 GBS serotypes

Five serotypes were determined in the present 200 isolates, and the frequencies were ranked from high to low as following: serotype Ib (69, 34.5%), serotype V(52, 26.0%), serotype III(43,21.5%), serotype Ia (4, 2.0%), and serotype VIII (2, 1.0%). In addition, 30 isolates (15.0%) were non-typable (NT) using the present typing method. The coverage rate of the hexavalent vaccine (Ia, Ib, II, III, IV, and V) currently in development is 84.5%. There is no difference in serotype distribution between males and females. However, the serotypes of GBS varied across different age groups, pregnant groups, and strains with clinical relevance. (**Table 2**). Serotypes Ib (31.5%) and V (32.3) were clearly predominant in the 18–39 year olds; in the 40–64 year olds, Ib (32.6%) and III (32.6%) were predominant; >64 years, Ib (50.0%) as well as NT (26.7%) were predominant. In pregnant women, serotype V (36.4%), Ib (28.0%) are the obviously dominant serotypes, and in the non-pregnant women, serotype Ib (45.8%) and III (27.1%) predominate. In addition, serotypes Ib (39.5%) and III (24.4%) were the primary clinical pathogenic isolates, whereas serotypes V (34.2%) and Ib (30.7%) predominated among the colonized isolates. Notably, the proportion of serotype V varies markedly, with 32.3% in the 18–39 age group versus 18.6% in the 40–64 age group and 10% in >64 age group (P = 0.019), 36.4% in pregnant women compared to 14.0% in non-pregnant women (P < 0.001), and 34.2% in colonized strains as opposed to 15.1% in infectious strains (P = 0.036).

**Table 2:** Serotype distribution of the present 200 GBS in different populations [n (%)].

Characteristic	*N*	Ia	Ib	III;	V	VIII	NT^c^	*P* values
Sex	200							0.721
Male	34	0 (0.0)	12 (35.3)	6 (17.6)	8 (23.5)	0 (0.0)	8 (23.5)	
Female	166	4 (2.4)	58 (34.7)	37 (22.2)	44 (26.3)	2 (1.2)	22 (13.2)	
Age (years)	200							0.019
18-39	127	2 (1.6)	40 (31.5)	26 (20.5)	41 (32.3)	1 (0.8)	17 (13.4)	
40-64	43	2 (4.7)	14 (32.6)	14 (32.6)	8 (18.6)	0 (0.0)	5 (11.6)	
>64	30	0 (0.0)	15 (50.0)	3 (10.0)	3 (10.0)	1 (3.3)	8 (26.7)	
Pregnancy^a^	166							<0.001
Pregnant	107	1 (0.9)	30 (28.0)	21 (19.6)	39 (36.4)	1 (0.9)	15 (14.0)	
Non-pregnant	59	3 (5.1)	27 (45.8)	16 (27.1)	5 (8.5)	1 (1.7)	7 (11.9)	
Clinical relevance	200							0.036^d^
Carriage	114	1 (0.9)	35 (30.7)	22 (19.3)	39 (34.2)	1 (0.9)	16 (14.0)	
Infectious^b^	86	3 (3.5)	34 (39.5)	21 (24.4)	13 (15.1)	1 (1.2)	14 (16.3)	
Invasive	16	0 (0.0)	3 (18.8)	5 (31.3)	3 (18.8)	0 (0.0)	5 (31.3)	
Total	200 (100.0)	4 (2.0)	69 (34.5)	43 (21.5)	52 (26.0)	2 (1.0)	30 (15.0)	–

Pregnancy^a^: only females are included; Infectious^b^: Includes both invasive and non-invasive infection isolates; NT^c^: non-typable; 0.036^d^: Comparisons were made between the serotype distribution of infected and colonized isolates.

**Figure 2:**
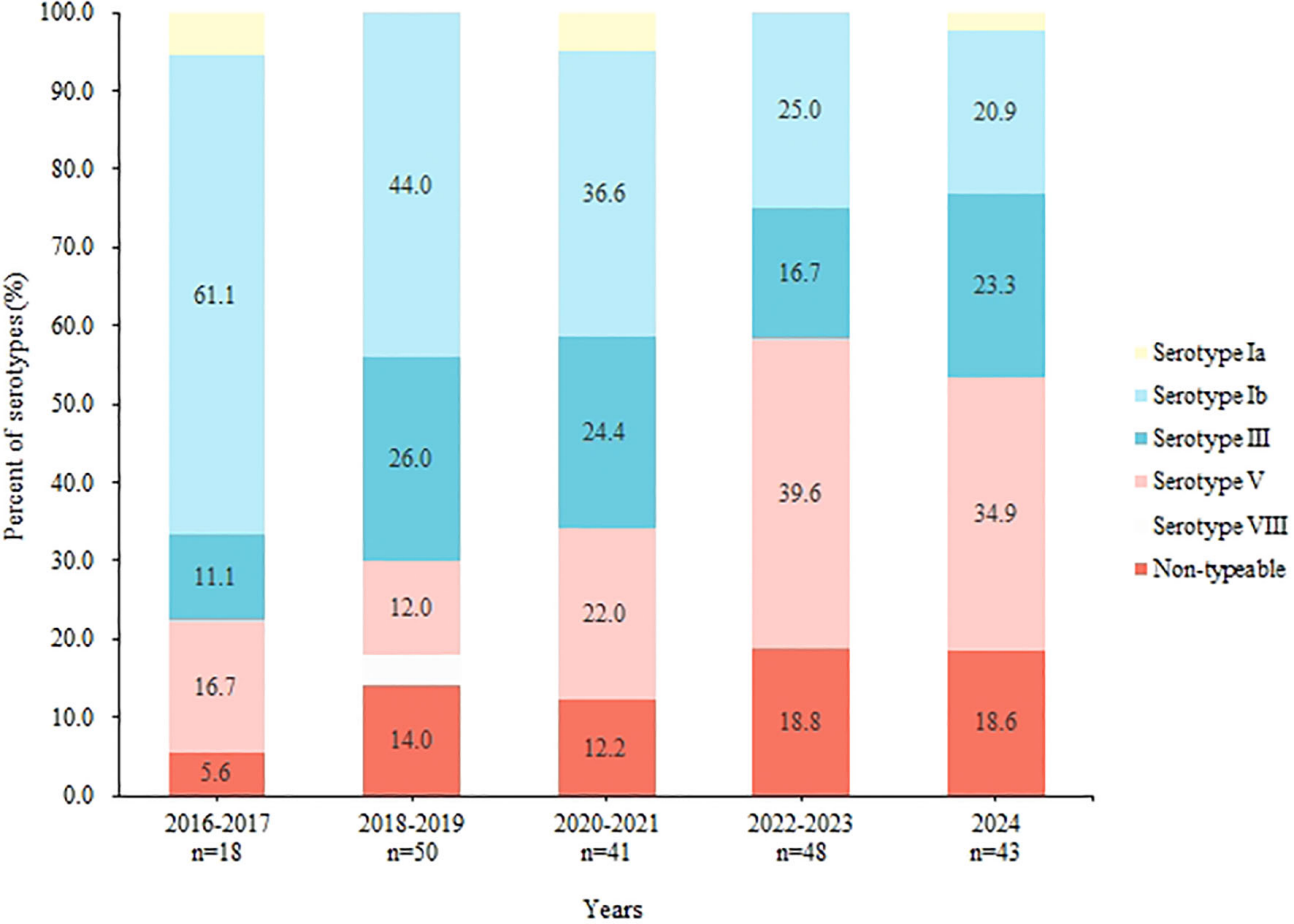
Changes in serotype proportions of GBS, 2016-2024.

During the study period, we observed significant temporal shifts in the composition of GBS serotypes. The proportion of serotype Ib decreased consistently from 61.1% in 2016–2017 to 20.9% in 2024 (P < 0.001). In contrast, the prevalence of serotype V and non-typable (NT) strains increased overall. Serotype III exhibited fluctuations within a range of 11.1% to 26.0% (**Figure 2**).

### 3.3 Antimicrobial Susceptibility

The results of antimicrobial susceptibility test against 20 antimicrobials of the present 200 GBS strains were shown in **Table 3**. Four bacterial strains exhibited penicillin resistance during automated testing, with MIC values ranging from 0.5 mg/L to 2.0 mg/L. However, subsequent E-test analysis revealed MIC values of 0.06 mg/L to 0.12 mg/L, indicating susceptibility to penicillin. All 200 GBS strains were sensitive to penicillin, and the resistance rates to erythromycin, azithromycin, levofloxacin, clindamycin, tetracycline, and chloramphenicol were 93.5%, 93.5%, 70.5%, 70.0%, 53.5%, and 13.0%, respectively (**Table 3**).

**Table 3:** Antimicrobial susceptibility against 20 antimicrobials of the present 200 GBS strains [n (%)].

Antimicrobial	S^a^	I^b^	R^c^	MIC_50_^d^ (mg/L)	MIC_90_^e^ (mg/L)	MICrange (mg/L)
Penicillin	200 (100.0)	–	–	0.06	0.06	≤0.03-0.12
Cefotaxime	200 (100.0)	–	–	≤0.12	≤0.12	≤0.12-0.25
Cefepime	200 (100.0)	–	–	≤0.05	≤0.05	≤0.25-≤0.05
Ceftriaxone	200 (100.0)	–	–	≤0.12	≤0.12	≤0.12-0.5
Cefuroxime (sodium)	–	–	–	≤0.05	≤0.05	≤0.05-1
Amoxicillin/clavulanate	–	–	–	≤2	≤2	<2-≤2
Ertapenem	200 (100.0)	–	–	≤0.5	≤0.5	≤0.5
Meropenem	200 (100.0)	–	–	≤0.25	≤0.25	≤0.25-0.5
Vancomycin	200 (100.0)	–	–	≤0.05	≤0.05	≤0.05-1
Daptomycin	200 (100.0)	–	–	0.5	0.5	≤0.06-1
Erythromycin	13 (6.5)	0 (0.0)	187 (93.5)	>2	>2	≤0.25->2
Azithromycin	13 (6.5)	0 (0.0)	187 (93.5)	>2	>2	≤0.25->2
Tetracycline	91 (45.5)	2 (1.0)	107 (53.5)	>8	>8	≤1->8
Tigecycline	–	–	–	0.03	0.03	≤0.015-0.12
Levofloxacin	56 (28.0)	3 (1.5)	141 (70.5)	>4	>4	≤0.05->4
Moxifloxacin	–	–	–	4	4	≤1-8
Chloramphenicol	174 (87.0)	1 (0.5)	25 (13.0)	4	32	2->32
Clindamycin	60 (30.0)	4 (2.0)	136 (70.0)	>1	>1	≤0.12->1
Linezolid	200 (100.0)	–	–	1	2	0.5-2
Cotrimoxazole	–	–	–	≤0.05	≤0.05	≤0.05

S^a^: susceptible; I^b^: intermediate; R^c^:resistance; MIC_50_^d^: 50th percentile of MIC values; MIC_90_^e^: 90th percentile of MIC values

**Figure 3:**
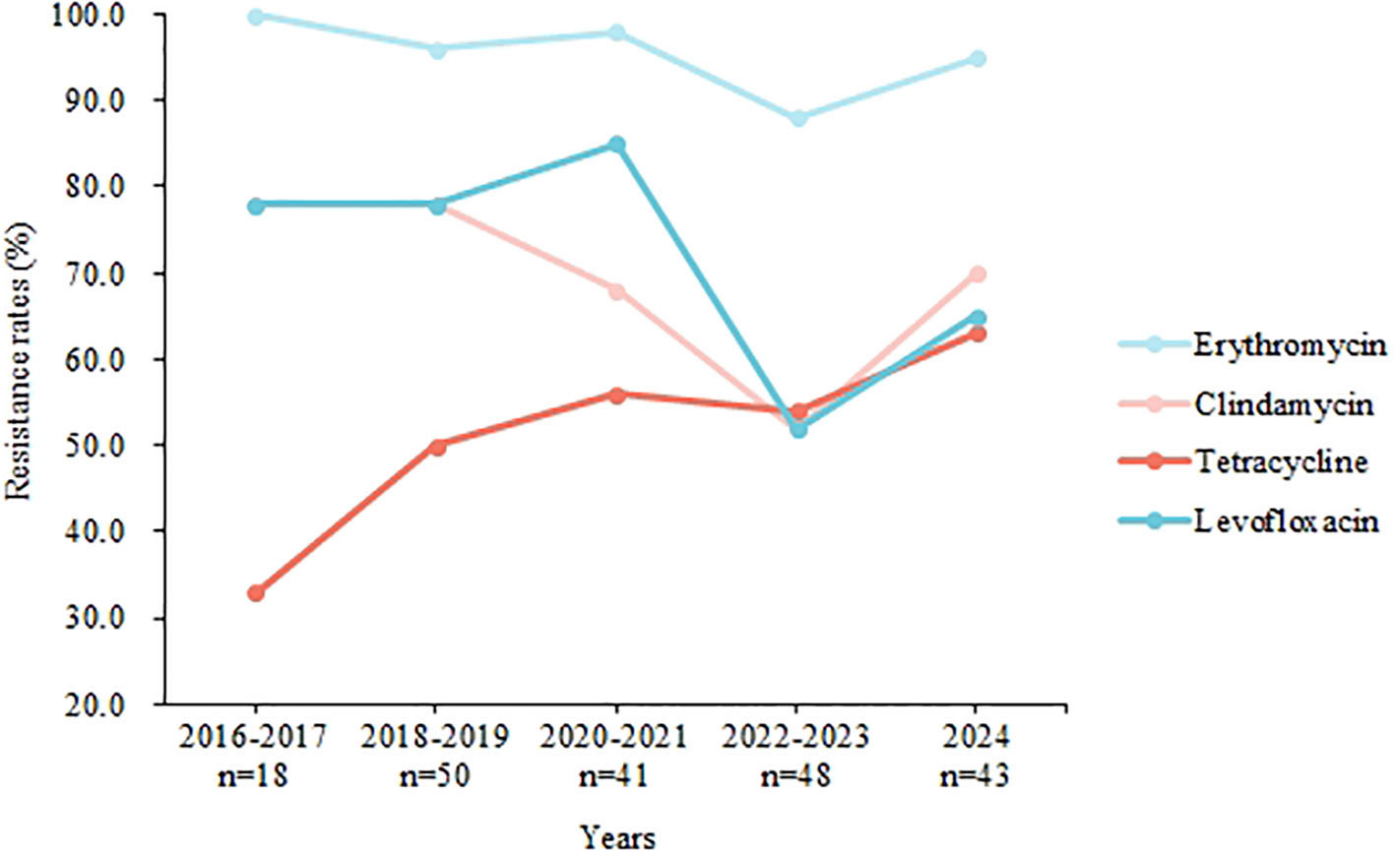
Trends in the present GBS isolate resistance to our antimicrobials, 2016-2024.

When comparing resistance rates among several common serotypes, significant differences were observed for erythromycin, azithromycin, levofloxacin, clindamycin, and tetracycline (**Table 4**). For instance, serotype Ib exhibited a tetracycline resistance rate of 10.0%, whereas serotype III showed resistance in 90.7% of cases (P < 0.001). Additionally, serotype Ib had a levofloxacin resistance rate of 92.9%, compared to 44.2% in serotype V (P < 0.001). **Figure 3** illustrates the yearly trends in resistance rates of GBS isolates to erythromycin, clindamycin, tetracycline, and levofloxacin. While resistance rates for erythromycin, clindamycin, and levofloxacin fluctuated over the years without a clear overall pattern, the resistance rate to tetracycline showed a gradual increase. Specifically, the tetracycline resistance rate was 33.3% in 2016–2017 but rose to 62.5% by 2023 (P = 0.056).

**Table 4:** Comparison of antimicrobial resistance rates among the common GBS serotypes [n (%)].

Serotype	Erythromycin	Azithromycin	Clindamycin	Tetracycline	Levofloxacin
Ib (n=69)	67 (97.1)	67 (97.1)	65 (94.2)	6 (8.7)	65 (94.2)
V (n=52)	50 (96.2)	50 (96.2)	19 (36.5)	45 (86.5)	23 (44.2)
III; (n=43)	35 (81.4)	35 (81.4)	27 (62.8)	39 (90.7)	31 (72.1)
NT (n=30)	30 (100.0)	30 (100.0)	21 (70.0)	14 (46.7)	20 (66.7)
Others^a^ (n=6)	1 (16.7)	1 (16.7)	4 (66.7)	3 (50.0)	2 (66.7)
*P* values^b^	0.001	0.001	<0.001	<0.001	<0.001

Others^a^: serotype Ia, VIII; *P* values^b^: only compared the resistance rates of serotype Ib, III, V, and NT.

## 4 Discussion

This study encompassed 200 GBS isolates from adults collected over a 9-year period at a single hospital. GBS colonization and infection were identified across all age groups and genders, with 18.6% of strains causing invasive GBS infections in adults (**Table 1**). However, in most regions of China, screening efforts are currently limited to pregnant women, which may lead to underestimation of GBS colonization rates in other adult populations. The findings indicate that GBS infection and colonization are relatively common among adults of all ages, comparable to the occurrence in pregnant women. In a 24-year Norwegian study, 3710 invasive GBS infections were identified from non-pregnant adults, and the median age of patients was 68 years (IQR 53-79), and 49.7% of cases were male (Uggen et al., 2024). In Italy, from 2015 to 2019, a total of 108 GBS were collected from non-pregnant patients with a mean age of 70.8 years (range: 29–97 years), of which 63.1% were male (Imperi et al., 2024). Unfortunately, clinical and scientific research often overlook cases of GBS infection and colonization in non-pregnant adults. In particularly, previous studies on GBS infection in China have mainly focused on pregnant women and newborns. The prophylactic use of prenatal antibiotics has proven effective in preventing GBS infection in perinatal women and EOD in newborns; however, it shows limited efficacy to prevent GBS-LOD (Russell et al., 2017). Prior research has established that premature birth, low birth weight, and maternal colonization with GBS are the primary risk factors for GBS-LOD (Karampatsas et al., 2022). However, few studies have assessed the potential impact of GBS colonization by other adults in the mother and newborns’ environment. This may be an obstacle to developing effective prevention and control strategies against GBS-LOD in neonates.

Among the 200 GBS strains isolated in this study, serotype Ib was the predominant serotype (34.5%), followed by serotype V (26.0%) and III (21.5%), whereas serotype Ia was isolated at a lower rate (2.0%), while NT was isolated at a higher rate (15.0%) (**Table 2**). This serotype distribution shows a significant difference compared to the rates observed in pregnant women from regions such as Beijing (III-41.7%, V-18.8%, n=96), Xiamen (III-55.0%, Ib-16.4%, n=298), and Xi’an (III-43.3%, Ia-18.7%, n=150) in China (Li et al., 2018; Lin et al., 2020; Xie et al., 2019). However, the results are like those reported by Gong et al (Gong et al., 2024). for non-pregnant adults with GBS infection in the Shandong region, where serotypes Ib accounted for 48.2% and serotype III for 24.5% of the cases (n=139). These differences could be attributed to a multitude of factors, including geography, the population source of strains, the site of isolation, and temporal variations. Despite the significant variations in GBS types observed among the studies in China, the common types identified across all studies are largely consistent, encompassing type Ia, Ib, III and V. These types are encompassed within the scope of vaccines currently being developed against six capsular polysaccharide (Madhi et al., 2023), resulting in a vaccine coverage rate ranging from 93.6% to 100.0% in these studies. This vaccine has a coverage of up to 84.5% in the present isolates. These high vaccine coverage rates indicate a promising preventive effect for future vaccinations. However, a major vaccine-related concern is the possibility of capsular switching among GBS (Bellais et al., 2012), it remains difficult to predict what will be the impact of a CPS vaccine on GBS population. We should always conduct continuous strain surveillance to ensure the long-term effectiveness of vaccines. Besides, we observed that the distribution of GBS serotypes varies across different age groups (**Table 2**). This variation may be related to the host’s immune status (Kim et al., 2024). For instance, a study in South Korea showed that serotype VIII has emerged as a predominant pathogenic serotype among non-pregnant adults, particularly in those aged over 65 years (Kim et al., 2024). Pregnancy status can also alter the host’s immune state, thereby influencing GBS colonization and infection (Chinese Society of Perinatal Medicine, Obstetrics Group of Chinese Society of Obstetrics and Gynecology, 2021). These findings highlight the importance of understanding the distribution of GBS serotypes across diverse populations to inform targeted prevention strategies and vaccine development.

This research identified a temporal shift in the diversity of GBS serotype distribution (**Figure 2**). Specifically, there was a notable decrease in the prevalence of serotype Ib, from 61.1% between 2016 and 2017 to 20.9% in 2024 (P < 0.001). This findings contrast with that of Lopes, who reported that serotype Ib became the predominant serotype among non-pregnant adults in Portugal after 2013 (Lopes et al., 2018). And in America, between 2008 and 2016, the incidence of serotype Ib disease doubled from 0.8 to 1.6 cases per 100 000 (Francois Watkins et al., 2019). Additionally, we observed that serotype Ib exhibited a significantly lower resistance rate to tetracycline (5.6%) compared to other serotypes. Tetracycline is generally not used to treat GBS infections but is commonly employed in livestock, with consumption reaching 10,002.73 tons, exceeding global levels (Zhao et al., 2023). Research by Zhang et al. indicates that GBS resistance to tetracycline is often associated with specific tetM and tetO genes, which Ib/ST10 strains typically do not carry (Zhang et al, 2021). Given this, we speculate that elevated levels of tetracycline in the environment may account for the reduced isolation rate of serotype Ib (Sørensen et al., 2019). This hypothesis also explains the observed upward trend in tetracycline resistance rates in our study (**Figure 3**).

All the 200 GBS isolates were susceptible to β-lactam antibiotics, including penicillins, cephalosporins, and carbapenem antibiotics, which is in line with the current preferred recommendations for the prevention and treatment of GBS infections in China (Chinese Society of Perinatal Medicine, Obstetrics Group of Chinese Society of Obstetrics and Gynecology, 2021). Besides this, our study revealed that GBS demonstrated significant resistance to erythromycin, clindamycin, and levofloxacin. The resistance rate to levofloxacin, in particular, stood at 70.5%, which is markedly higher than the rates reported in Chinese neonates and pregnant women (Wang et al., 2023). This elevated level of resistance is a concerning development and emphasizes the necessity for judicious use of antibiotics and continuous monitoring to inform and refine clinical treatment strategies. This high resistance to quinolones is likely due to the broad use of these drugs in treating urinary and gastrointestinal tract infections in adults. However, such medications are generally not recommended for use in neonates due to potential adverse effects on the skeletal and muscular systems (Li et al., 2022).

This study acknowledges certain limitations. First, as a single-center investigation, our bacterial samples were exclusively sourced from the Baoji region in China. This geographical restriction may limit the generalizability of our findings to the broader national context of adult GBS infections. Second, the lack of detailed antibiotic treatment histories for the patients introduces potential biases in our assessment of GBS strain resistance patterns. Third, the retrospective nature of the study resulted in incomplete clinical data, particularly regarding underlying diseases in some patients. This limitation precludes a more nuanced evaluation of the relationship between GBS disease and associated comorbidities. Despite these limitations, the key findings of this study remain robust. It unequivocally establishes the presence of GBS infection and colonization across all age groups in Baoji’s adults, China. Furthermore, it provides valuable insights into GBS infection and colonization in males, as well as invasive infections in adults. These findings have significant implications for shifting the clinical and research focus from perinatal GBS infections to a broader understanding of adult GBS epidemiology. Additionally, the serotype and resistance profiles of the strains identified in this study offer a foundational reference for future large-scale investigations, highlighting the characteristics of adult-specific and invasive strains.

## 5 Conclusion

GBS can infect and colonize individuals of all genders and age groups in Baoji, China. Serotypes Ib, III, and V are the most common, yet their frequency varies with host age, pregnancy status, and clinical context. The distribution of serotype diversity has shifted significantly over time, with serotype III now emerging as the most frequent. Although GBS isolates generally remain susceptible to penicillin, resistance patterns differ among serotypes. Continuous surveillance of GBS resistance patterns is crucial for guiding clinical management and informing public health strategies.

## Data Availability

The original contributions presented in the study are included in the article/supplementary material. Further inquiries can be directed to the corresponding author.
